# Enhancing Home Health Mobile Phone App Usability Through General Smartphone Training: Usability and Learnability Case Study

**DOI:** 10.2196/humanfactors.7718

**Published:** 2018-04-26

**Authors:** Richard Harte, Tony Hall, Liam Glynn, Alejandro Rodríguez-Molinero, Thomas Scharf, Leo R Quinlan, Gearóid ÓLaighin

**Affiliations:** ^1^ Electrical & Electronic Engineering School of Engineering & Informatics NUI Galway, University Road Galway Ireland; ^2^ Human Movement Laboratory NUI Galway University Road Galway Ireland; ^3^ CÚRAM Centre for Research in Medical Devices NUI Galway, University Road Galway Ireland; ^4^ School of Education NUI Galway University Road Galway Ireland; ^5^ General Practice School of Medicine NUI Galway, University Road Galway Ireland; ^6^ Clinical Research Unit Consorci Sanitari del Garraf Sant Pere de Ribes Barcelona Spain; ^7^ Irish Centre for Social Gerontology Institute for Lifecourse and Society NUI Galway, University Road Galway Ireland; ^8^ Physiology School of Medicine NUI Galway Galway Ireland

**Keywords:** smartphone, aged, elderly, wearable electronic devices, telemedicine, user-computer interface, education, user centered-design, usability, connected health, human factors, falls detection

## Abstract

**Background:**

Each year, millions of older adults fall, with more than 1 out of 4 older people experiencing a fall annually, thereby causing a major social and economic impact. Falling once doubles one’s chances of falling again, making fall prediction an important aspect of preventative strategies. In this study, 22 older adults aged between 65 and 85 years were trained in the use of a smartphone-based fall prediction system. The system is designed to continuously assess fall risk by measuring various gait and balance parameters using a smart insole and smartphone, and is also designed to detect falls. The use case of the fall prediction system in question required the users to interact with the smartphone via an app for device syncing, data uploads, and checking system status.

**Objective:**

The objective of this study was to observe the effect that basic smartphone training could have on the user experience of a group that is not technically proficient with smartphones when using a new connected health system. It was expected that even short rudimentary training could have a large effect on user experience and therefore increase the chances of the group accepting the new technology.

**Methods:**

All participants received training on how to use the system smartphone app; half of the participants (training group) also received extra training on how to use basic functions of the smartphone, such as making calls and sending text messages, whereas the other half did not receive this extra training (no extra training group). Comparison of training group and no extra training group was carried out using metrics such as satisfaction rating, time taken to complete tasks, cues required to complete tasks, and errors made during tasks.

**Results:**

The training group fared better in the first 3 days of using the system. There were significant recorded differences in number of cues required and errors committed between the two groups. By the fourth and fifth day of use, both groups were performing at the same level when using the system.

**Conclusions:**

Supplementary basic smartphone training may be critical in trials where a smartphone app–based system for health intervention purposes is being introduced to a population that is not proficient with technology. This training could prevent early technology rejection and increase the engagement of older participants and their overall user experience with the system.

## Introduction

### Background

Digital mobile telephony potentially creates new opportunities to augment health care. Owing to their interactive features, large storage capacity, communication capabilities, and ability to access large knowledge databases, smartphones can present a novel means to deliver health care to individuals in the home. Consequently, smartphones are being used to deliver an increasingly wide range of personal health care solutions [1,2]. Although older adults have traditionally adopted new technology at lower rate than other age cohorts, the Pew Internet Research Center reports that the use of Internet technology by older adults is steadily increasing, with 2012 being the first year where more than half of people in the United States aged 65 years and older were using the Internet [3]. Recent studies have shown that older adults have a rich technology profile in terms of home appliances, TVs, PCs, and mobile phone apps and only differ from other technology using age groups in terms of Internet-based technology [4]. Although today’s older adults have better uptake of mobile technologies than previous older adult groups [5], contemporary mobile devices such as smartphones still present a substantial challenge for older adults [6]. These challenges can be a result of numerous factors such as unfamiliarity or fear of technology, lack of perceived usefulness (PU), lack of perceived ease of use (PEoU), diminished interactive capabilities, and poor usability characteristics of the devices in question [7,8].

Methodological steps can be taken during the design process for these devices to ensure that the demands of the device do not exceed the capabilities of the older adult user (ref). For example, steps can be taken to ensure that interface elements such as buttons and text are usable, the device navigation can be designed to ensure that basic tasks only require a small number of steps, and the supporting documentation can be presented in an intuitive and simple manner [9-11]. However, these design aspects may not mitigate a new user’s unfamiliarity with the device, and therefore, the potential for technology rejection may remain quite high [12]. This could have adverse effects when attempting to introduce smartphones to older adult users for the delivery of health care using an mHealth, telemedicine, or connected health infrastructure. In a previous study, a smartphone-based fall detection and prevention system was tested on a group of 39 older adult users over a 10-day period. Despite the system having undergone a full human-centered design process and the participants receiving adequate training on how to use the system, the system scored 70 of 100 on the System Usability Scale (SUS), indicating only average usability [13]. We suspected that unfamiliarity with smartphones and the specific demands of interaction with a smartphone, particularly the unique touch screen interactions required, may have led to poor usability outcomes. From this experience, we concluded that the outcomes of many trials and studies that involve the use of home health app design to run on smartphones could be compromised because of a lack of familiarity with the basic functioning of the device.

A period of pretrial introductory smartphone training in conjunction with concurrent recall-based learning tasks could present a potential solution to this problem. Effective training could provide a complete novice with a better chance of adopting the technology, thereby increasing the potential effectiveness of smartphone-based mHealth and connected health interventions for that person. In a study of a group of older adults who were being introduced to an mHealth pain management smartphone app, 61% of the participants cited “provide training on device use” as the main requirement if their potential use of the technology was to be enhanced and ultimately sustained, followed by 30% who cited “tailoring the device to the user’s functional needs (ie, usability)” as the secondary requirement [14]. Therefore, we can conclude that twice as many participants felt that adequate introductory training was more important than enhancements to the design of the app in terms of usability. With this important finding in mind, this paper will provide an enumerative and detailed methodology to achieve this introductory training as efficiently as possible and therefore potentially mitigate any potential usability problems the technology may have.

### Objectives

**Figure 1:**
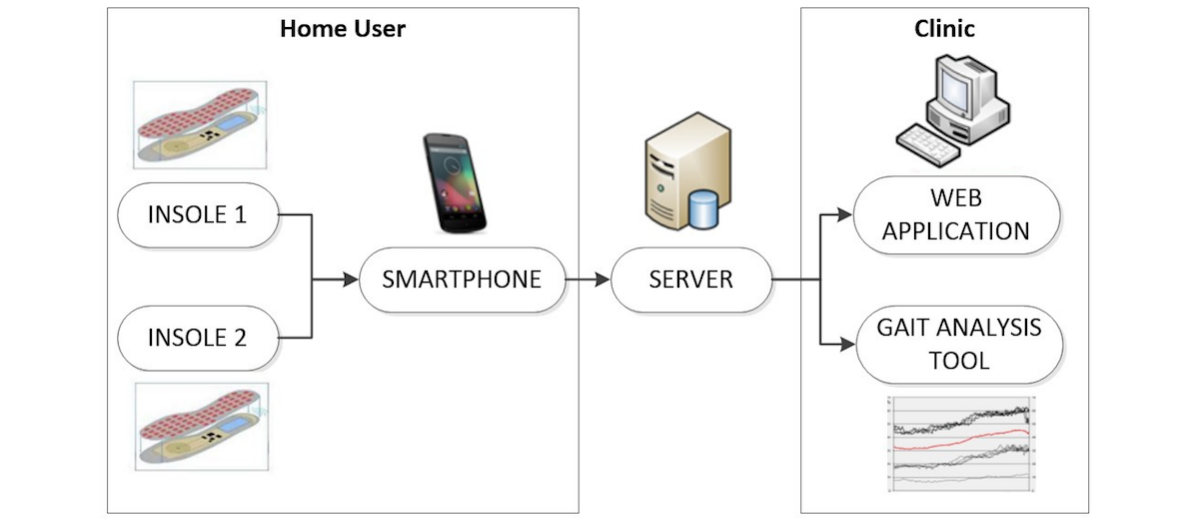
The Wireless Insole for Independent and Safe Elderly Living (WIISEL) System.

In this study, we trained 22 participants to use the same smartphone-based fall detection and prevention system, as described in the study by Stara et al [13], over a period of 5 days. This system is the Wireless Insole for Independent and Safe Elderly Living (WIISEL) system [15]. The system is designed to continuously assess fall risk by measuring various gait and balance parameters and is also designed to detect falls. The system is targeted at older adults who are at high risk of falling [16]. The system consists of a pair of instrumented insoles and a smartphone, which are worn by the user during daily activity. Data collected by embedded sensors in the insoles are sent to the smartphone and then uploaded via an Internet connection to a server in a clinic for processing and analysis. The data are presented in various ways to a specialist via a Web app and desktop-based gait analysis tool. The architecture of the system is illustrated in Figure 1.

The 22 participants were instructed to carry out a number of specific tasks with the WIISEL smartphone app (see the Methods section for details of these tasks). Half of the participants were provided with additional concurrent training in the general use of the smartphone, which began 2 days before the trial and continued. Our hypothesis was that the group that received the additional smartphone training would have a better user experience with the system.

## Methods

### Participants

Participants were recruited from the Galway city area. Twenty-two participants were recruited 74±5.5 years providing informed consent under ethical approval provided by University College Hospital Galway. Participants were split into 2 groups as outlined in Table 1. Whether a participant belongs to Group 1 (No extra training) or Group 2 (Extra training) was decided at random with 50% of participants belonging to each group.

All training was carried out in the participant’s home by the lead researcher who followed the same protocol for each participant. The researcher visited the participants’ home each day to teach them new tasks and to observe them carrying out previously learned tasks. A systematic cuing hierarchy approach and a “think aloud” protocol [17] were used by the lead researcher for the training procedure. These will be outlined in greater detail.

### Technology Acceptance Indicators

To indicate how participants would fare with the introduction of the new technology, their current mobile technology capability was assessed. Participants were split into subgroups based on their previous experience with mobile technology to allow for further analysis. When it comes to classifying users based on their expertise or previous experience with mobile technology, there is no set classification system. Most so-called “expert” users are simply users who have gained hands-on experience of using the technology and may not have used a user manual for their device [18]. However, using an understanding of users’ prior technical knowledge to predict their future adoption of technology is well supported [19]. We classified the participants into 3 separate categories based on their observed performance in carrying of a series of simple functional tasks with their mobile device. The functional tasks were chosen as representing real-world use requirements of the mobile device and were sufficiently challenging to highlight differences in skill level among user groups [18]. The 3 categories are outlined in Table 2.

In this context, “feature phone” is a retronym used to describe low-end mobile phones which are limited in capabilities in contrast to smartphones, that is, they are phones that have basic call and short message service functionality but do not have extensive media or Internet capabilities**.**

This procedure for practically dividing participants into technology experience groups is illustrated in the flowchart in Multimedia Appendix 1.

### Perceived Ease of Use and Perceived Usefulness

PEoU and PU are the 2 key usability indicators pertaining to technology design. The influence of PEoU and PU on behavioral intent, and hence technology adoption, has been supported for the use of technology by older adults and specifically for the use of communication technology [20]. To measure whether there was any correlation between either PU or PEoU and the eventual usability outcomes, we measured the participant’s PEoU and PU of smartphone technology before the trial started. We used a 7-point Likert scale using items from the technology acceptance model [21,22] to establish PU and PEoU.

**Table 1:** Participants were split into 2 groups based on what kind of training they would receive.

Group	Label	Training
1	No extra training	Only receive smartphone training which is necessary to operate the WIISEL^a^ app. This group used the WIISEL system in their home for 5 days.
2	Extra training	This group also used the WIISEL system in the home for 5 days, but received extra smartphone training which began 2 days before starting the WIISEL trial and continued for the first three days of the WIISEL trial. The smartphone training was intended to make these participants familiar with the functions of the phone.

^a^WIISEL: Wireless Insole for Independent and Safe Elderly Living.

**Table 2:** Participants within each group were further classified based on their observed performance.

Category	Definition	Example
1 (novice user)	No experience with smartphones and basic capability with feature phone.	A user who does not own any sort of mobile phone OR a user who does own a feature phone but cannot demonstrate to satisfactory level that they can make a call, receive a call, and send or receive a text message without problems.
2 (intermediate or average tech user)	Perfect capability with feature phone, but limited capability with smartphones, may have been exposed to touch screen interfaces before.	A user who does own a feature phone and can demonstrate to a satisfactory level that they can make a call, receive a call, and send a text message OR a user who owns a smartphone and can demonstrate to a satisfactory level that they can make and receive a call but who cannot effectively send a text message.
3 (competent user or familiar with Internet technologies and mobile devices)	Adequate capability with (own) smartphone or with related touch screen devices (such as a tablet)	A user who owns a smartphone and can demonstrate satisfactorily that they can make or receive a call, receive a call, and send a text message OR a user who owns a tablet and can successfully send an email to the researcher.

### Training Procedure

Our training procedure was based on an approach known as the errorless fading of cues technique [23]. This technique involves reducing the cues on repetitive tasks until the user can complete the task without error. The overall training schedule for Group 1 (No extra training) and Group 2 (Extra training) are outlined in Table 3. Training blocks are broken up to ensure that the participants were not overburdened with new training. Overall, each participant in Group 1 was subject to 0.75 to 1 hour of training or testing time per day, whereas each participant in Group 2 was subject to 1.5 to 2 hours of training or testing time per day. This included regular breaks and the time taken for the researcher to record metrics after each task. The number of days of training was chosen to be long enough to give participants the best chance of achieving some sort of mastery [23] but short enough to allow for convenience in having participants and trainer available for consecutive days.

The WIISEL specific tasks which were to be carried out by both Group 1 (No extra training) and Group 2 (Extra Training) are listed in Table 4. These tasks were selected based on a use case analyses of the WIISEL system [24] and were split into 2 different lesson blocks to reduce the burden on the participant by implementing small measurable objectives [25].

**Table 3:** Training schedule for each group. O indicate an introductory lesson, whereas X boxes indicate observational cue-assisted training. The dash (—) indicates no training on that day for that lesson block.

Group	Training type	Lesson block	Day −2	Day −1	Day 0	Day 1	Day 2	Day 3	Day 4	Day 5
1 (No extra training)	WIISEL^a^ app training	Block 1	—	—	O	X	X	X	X	X
		Block 2	—	—	—	O	X	X	X	X
2 (Extra training)	Smartphone training	Block 1	O	X	X	X	X	X	—	—
		Block 2	O	X	X	X	X	X	—	—
		Block 3	—	O	X	X	X	X	—	—
	WIISEL app training	Block 1	—	—	O	X	X	X	X	X
		Block 2	—	—	—	O	X	X	X	X

^a^WIISEL: Wireless Insole for Independent and Safe Elderly Living.

**Table 4:** Wireless Insole for Independent and Safe Elderly Living (WIISEL) tasks.

Lesson block	Task number	Task title	Task description
1	1	Check System Status	Turn on phone from power-off state, enter WIISEL^a^ app, and view system status
1	2	Connection Sequence	Enter app and carry out Connection Sequence to the WIISEL insoles
1	3	Upload Sequence	Upload WIISEL data from the app to the server
1	4	Minimize App	Minimize the app using the home button
1	5	Fall Detection Response	Respond to the fall alarm according to manual instructions
2	6	Reset Sequence	Carry out the app Reset Sequence via the smartphone settings
2	7	Log-in Sequence	Log-in to the app using the supplied username and password

^a^WIISEL: Wireless Insole for Independent and Safe Elderly Living.

Both groups were exposed to the WIISEL system over a course of 6 days (day 0 was training only, Days 1 to 5 were used to record performance, there was also some training on day 1, see step 2 in the following). This exposure consisted of the following steps:

1. On day 0 (first day of training), the researcher carried out a walkthrough for all the tasks in lesson block 1 (Table 4). For each task, the researcher walked the participant through the task and demonstrated it using the WIISEL user manual(s) as a reference (Multimedia Appendix 2). The participant repeated each task until no further cues were required. There was no recording of metrics on this day.
2. On day 1 (second day of training and first day of testing), the researcher carried out a walkthrough for all the tasks in lesson block 2 (Table 4), going through the same training routine as with lesson block 1 on day 0. The researcher also asked the participant to carry out the tasks in lesson block 1 by recalling their lessons from the previous day. The participant was instructed to try and complete the block 1 tasks without cues or input from the researcher, with the researcher providing cues only when it was clear that a cue was needed. The participant carried out each task 3 times.
3. At the end of each completed block 1 task, the user provided satisfaction ratings using the After Scenario Questionnaire (ASQ) [26]. Usability metrics such as task completion time, number of errors made, and number of cues required were recorded. The cuing hierarchy provided by the trainer comprised 5 different cue classifications [23], with 4=full explanation and demonstration; 3=the same verbal explanation as above but pointing to the next step before the participant executing it; 2=no verbal guidance provided, only pointing to the correct response before the participant executing it; 1=confirmation of a correct query, for example, “Do I tap details?”; and 0=no support provided. A lower score indicates greater proficiency with the device. Participants were given the standard instruction to think aloud [27].
4. From day 2 and day 5 on a daily basis, the participant carried out the WIISEL tasks from block 1 and block 2 (Table 4) under observation by the researcher. The researcher used the same metrics from day 1 (see step 3 above) to measure performance.
5. At the end of day 5, a semi-structured interview was carried out with each participant, and they also filled out the SUS questionnaire to provide an overall score of their user experience with the WIISEL system [28].

An example of the user manual for the WIISEL app is shown in Multimedia Appendix 2 and was the product of a comprehensive human-centered design process which tested and informed design changes of the WIISEL smartphone interface and the layout and content of the manual. Both Group 1 and Group 2 used this manual. Group 2 was also provided with smartphone-specific training in parallel with their WIISEL training. The smartphone-specific tasks are listed in Table 5. The smartphone tasks were selected based on recent studies on most popular usage patterns for smartphones [29,30].

This training was started 2 days before the WIISEL exposure began (we will refer to these days as day −2 and day −1). The routine was completed as follows:

1. On day −2, lesson blocks 1 and 2 (Table 5) were completed. For each task, the researcher walked the participant through the task and demonstrated it, using the NEXUS user manual(s) as a reference. The participant repeated each task until no further cues were required. There was no recording of metrics on this day.
2. On day −1, lesson block 3 (Table 5) was completed. Also on this day, the researcher asked the participant to try and recall their tasks from the previous day. The same recording of metrics was carried out as for the WIISEL training. This continued up to day 3.
3. It was seen as essential to gradually embed the task in its natural context with regular time constraints and less predictable occurrence [18]. Therefore, participants were asked to ring the lead researcher each day at a pre-agreed time and to send a short message service to accompany the in-situ observations [23].

The LG-Nexus 5 User Manual outlined in a step-by-step format how to complete basic tasks such as making phone calls and sending text messages (Multimedia Appendix 3). This manual was used for the Group 2 smartphone training.

**Table 5:** Smartphone tasks.

Lesson block	Task number	Task	Notes
1	1	Power and lock settings	Turn on phone from power-off state/unlock phone/lock phone
1	2	Dial a number to call	Dial a number into your phone and call it (present an arbitrary number)
1	3	Phone call	Receive a phone call and hang up and reject a phone call
2	4	Store a number in your phone and then call the stored number	Store a number in your phone, go to contacts to call the stored number
2	5	Text message	Read a text message/reply to the text message
3	6	Install an app	Install a television channel player (or similar) on the phone
3	7	Google search	Search for the term “Cinema Times Galway” in Google
3	8	Camera	Take a picture with the camera and go to gallery to see where the picture is stored

### Analyses of Data

Data were interrogated using both qualitative and quantitative approaches. To compare how each group performed in terms of the WIISEL tasks (Table 4), *t* tests were used to seek statistical significance between groups for metrics such as errors made, cues required, completion time, and ASQ scores for each task. To reduce the effects of potential outliers for the task completion time metric (eg, a participant takes an unusually long time because of very slow typing or has to return to the beginning of the task because of a serious error), the logarithmic-based geometric mean was used [31]. Mean SUS scores were also compared for each group using *t* tests. Ethnographic observations were also made on the types of errors committed with the smartphone by reviewing notes and videos of the lessons.

## Results

### Overview

The results are presented in a series of stages. First, we will present our findings on the technology profiles of the participants. Next, we will compare the individual metrics of task times, errors made, cues required, and ASQ score between the two groups for each WIISEL task. Next, we will compare the SUS score for the two groups from their use of the WIISEL system. Finally, we will compare the results with some other usability studies that have been carried out with the WIISEL system.

### Technology Profiles

The breakdown of participants into the different technology categories and age categories are presented in Table 6. The only significant correlation that was observed indicated that users with greater experience with mobile technology had a higher level of PU of smartphones. There was a minor correlation observed between increased technology experience and PEoU of smartphones. No significant correlation was found between age and PEoU (*R*=.2, *P*=.21) or between age and PU (*R*=−.04, *P*=.86). Weak positive correlation was found between technology experience and PEoU (*R*=.39, *P*=.15); Strong positive correlation was found between increased technology experience and PU (*R*=.6, *P*=.005).

Table 6 shows the results of the PE and PEoU questionnaire applied to each participant before the study began. The scale runs from 1 to 7, with 7 indicating a higher level of PU or PEoU. It provides a summary of correlation analysis between these questionnaires and age and technology categories of the participants.

In terms of mobile technology ownership, only 3 of the 22 participants (13%) did not own any sort of phone, whereas 6 (27%) owned a smartphone. The rest of the participants (60%) owned feature mobile phones, with the Nokia feature mobile phone (various models) proving the most popular. Five participants owned tablets although only 2 could be said to be proficient and frequent users. Of the 5 tablet users, none owned smartphones (all owned feature mobile phones). Therefore, the number of participants with some sort of touch screen experience was measured at 11 (50%).

### Group Comparison of Wireless Insole for Independent and Safe Elderly Living Tasks

#### Task Completion Time

**Figure 2:**
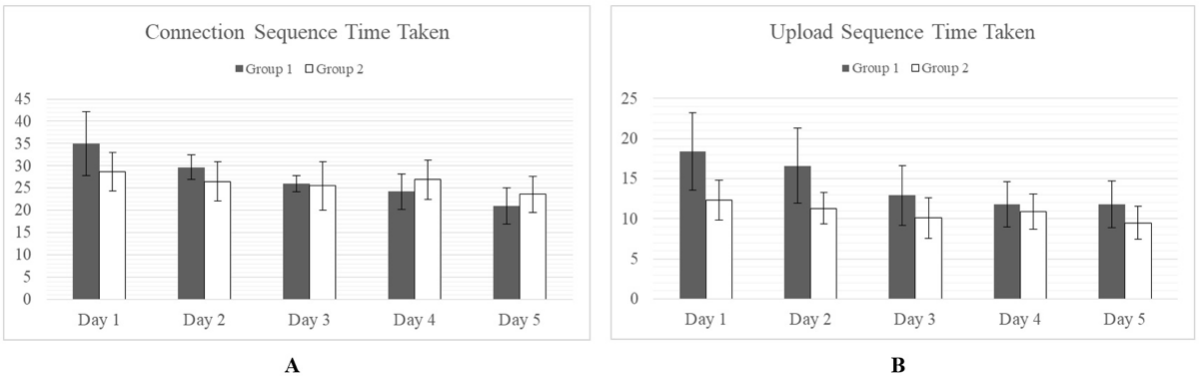
No significant difference in mean task completion time between Group 1 and Group 2 was observed for the Connection Sequence (A) or the Upload Sequence (B). All times are shown in seconds.

**Figure 3:**
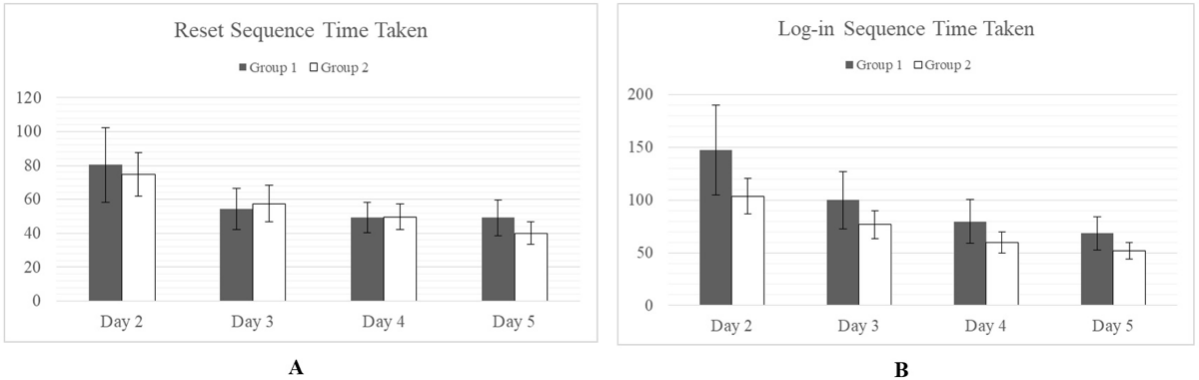
No significant difference in mean task completion time between Group 1 and Group 2 was observed for the Reset Sequence (A) or the Log-in Sequence (B). All times are shown in seconds.

Although, in general, Group 1 took longer to complete tasks, particularly on days 1-3, none of these differences were found to be statistically significant (alpha=.05). It was observed that there was much wider variance in the task times for Group 1 than Group 2 (Figures 2 and 3), particularly for the early days of testing. By days 4 and 5, however, variances were relatively equal, and the difference in means was negligible.

#### Cues Required to Complete Task

**Figure 4:**
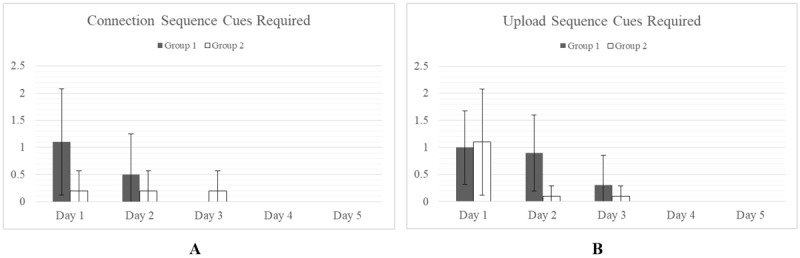
Cues required are measured as the total number of cue scores accumulated over the course of a task. No significant difference in cues required for Group 1 and Group 2 was observed for the Connection Sequence (A) or the Upload Sequence (B).

The cues required for the routine tasks such as Connection Sequence and Upload Sequence were negligible for each group with no significant differences found (Figure 4).

**Table 6:** Number of participants who fell into each different age groups and technology experience categories (based on demonstration).

Variable		Number of participants (n=22)	PEoU^a^ of smartphones	PU^b^ of smartphones
Mean score		—	5.3	5
**Age, in years**				
	65-69	5	5.3	4.7
	70-74	7	5.2	5.1
	75-79	5	4.7	5.6
	80+	5	5	6
**Technology category**				
	1 (novice)	6	4.4	4.5
	2 (intermediate)	10	4.9	5.2
	3 (expert)	6	5.6	6.3

^a^PEoU: perceived ease of use.

^b^PU: perceived usefulness.

**Figure 5:**
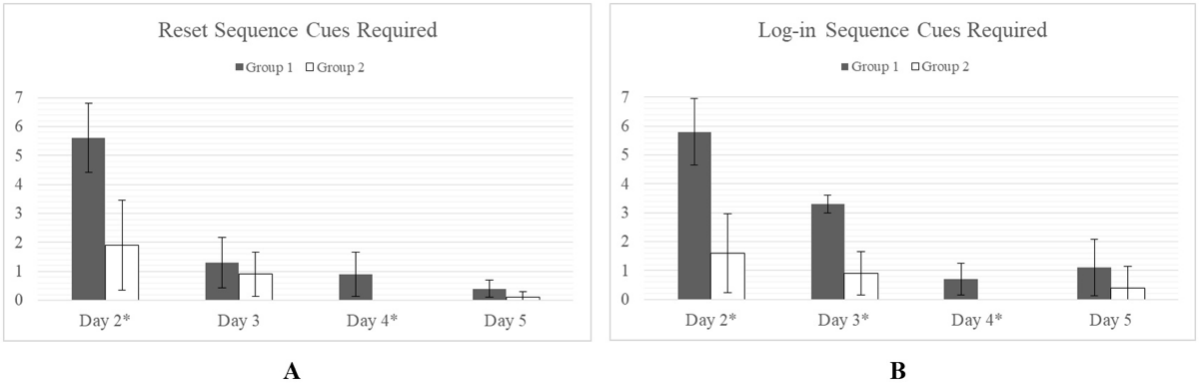
Cues required are measured as the total number of cue scores accumulated over the course of a task. *P<.05. Statistically significant differences are observed between Group 1 and Group 2 for the more complex Reset Sequence (A) and Log-in Sequences (B). By day 5, however, no difference is observed between each group.

However, significant differences (alpha=.05) in cues required were observed for the more complex tasks, Reset and Log-in Sequences. Group 1 required more cues on average than Group 2 for the Reset and Log-in Sequences occurring on days 2 and 3 (Figure 5). By day 5, both groups had reached parity.

#### Errors Committed During Each Task

**Figure 6:**
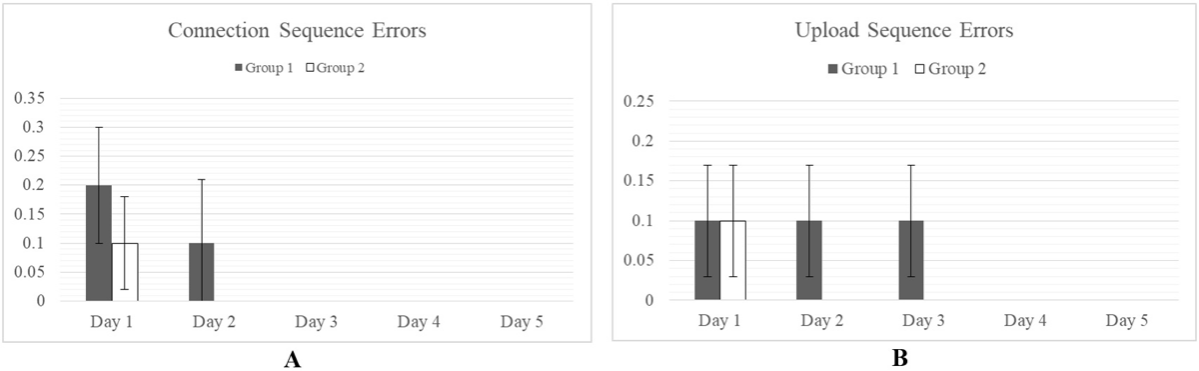
Average errors committed over the course of completing each task. No significant difference in errors committed between Group 1 and Group 2 was observed for the Connection Sequence (A) or the Upload Sequence (B).

Errors were counted as when a user reached a point in a task where they could not continue without carrying out either a reversing action or required them to start the task again. No significant difference was observed between groups during the Connection Sequence and Upload Sequence (Figure 6).

**Figure 7:**
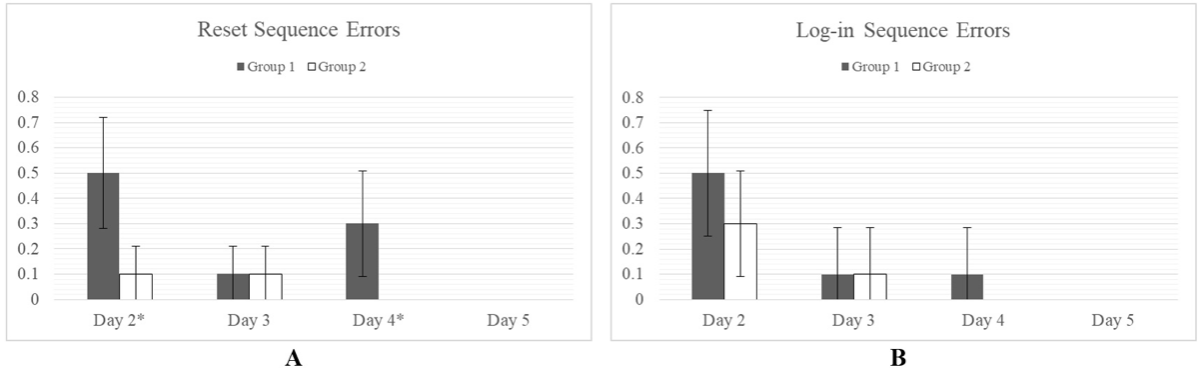
Average errors committed over the course of completing each task, *P<.05. Statistically significant differences are observed between Group 1 and Group 2 for the Reset Sequence (A). By day 5, no difference is observed between groups for any task.

Significant differences were observed. Statistically significant differences were observed between Group 1 and Group 2 for the Reset Sequence but not for the Log-in Sequence (Figure 7). By day 5, no difference was observed between groups for any task.

#### After Scenario Questionnaire Scores

**Figure 8:**
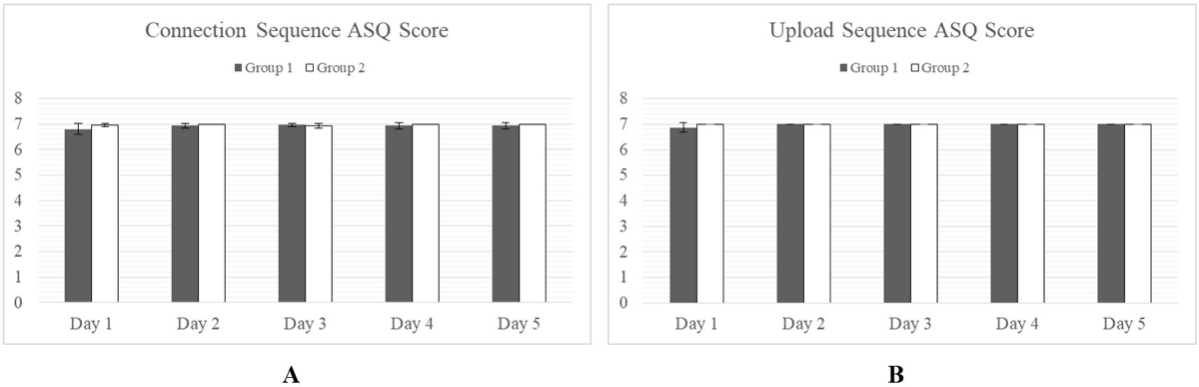
Satisfaction with the task in terms of ease, time taken, and supporting documentation. *P<.05. A score of 7 indicates maximum. No differences were observed for the Connection Sequence (A) or Upload Sequence (B) although significant differences were observed for the Log-in Sequence on days 2 and 3. ASQ: After Scenario Questionnaire.

ASQ scores show close agreement between groups on the ease of the Connection, Upload, and Reset Sequences (Figure 8).

**Figure 9:**
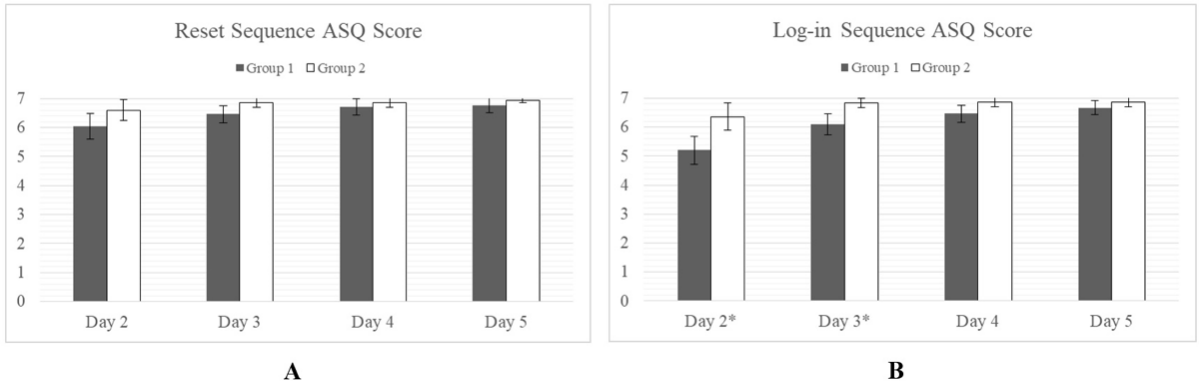
Satisfaction with the task in terms of ease, time taken, and supporting documentation. *P<.05. A score of 7 indicates maximum. No differences were observed for the Connection, Upload, and Reset Sequences although significant differences were observed for the Log-in Sequence on days 2 and 3. ASQ: After Scenario Questionnaire.

The only statistically significant (alpha*=*.05) difference was seen in days 2 and 3 of the Log-in Sequence when Group 1 was shown to have scored significantly lower than Group 2 (Figure 9). By day 4, both groups had reached parity.

### Overall User Experience

**Figure 10:**
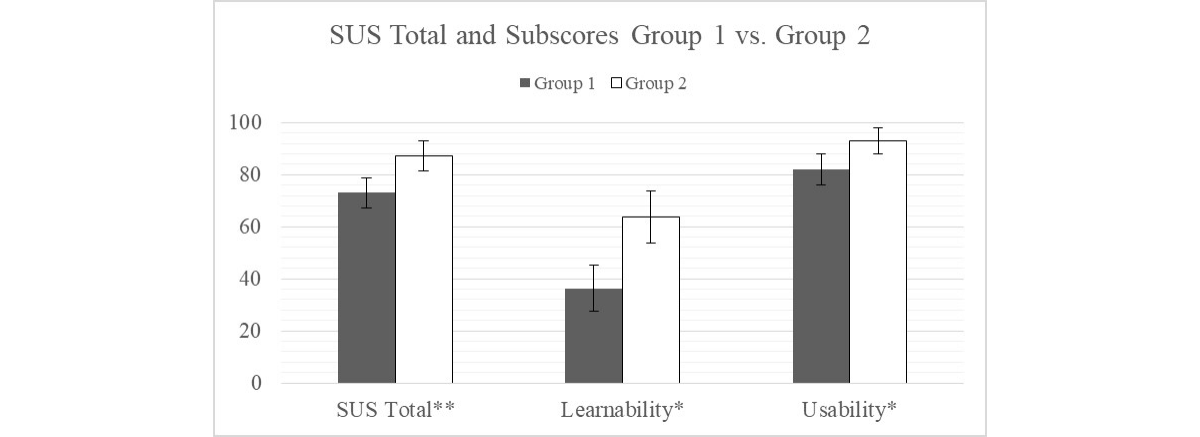
Satisfaction with the usability of the device. *P<.05, **P<.01. A score of 100 indicates maximum. Significant differences are observed between groups for each System Usability Scale (SUS) metric.

SUS scores showed significant differences (alpha=.05) between groups (Figure 10). In addition to measuring the total SUS, the scale was also split into its subscales to show learnability and usability scores. Group 1 versus Group 2 averages showed scores of 73 versus 87.25 (*P=*.007), 36.25 versus 63.75 (*P=*.04) and 82 versus 93 (*P=*.02) for SUS total, learnability, and usability, respectively.

#### Predictors of Positive User Experience

The regression analysis showed that there was a moderate to strong correlation between PEoU and PU and some of the SUS measures for each group. There was no significant correlation for age and technology experience as a predictor of SUS measures (Table 7).

#### The Impact of Performance Metrics on Overall User Experience

The regression analysis indicates that cues required, errors made, and the ASQ all influenced the SUS outcomes. Time taken was observed to be less of a factor, only showing strong correlation with learnability for Group 2 (Table 8).

### Comparison of Results With Previous Wireless Insole for Independent and Safe Elderly Living Usability Studies

**Figure 11:**
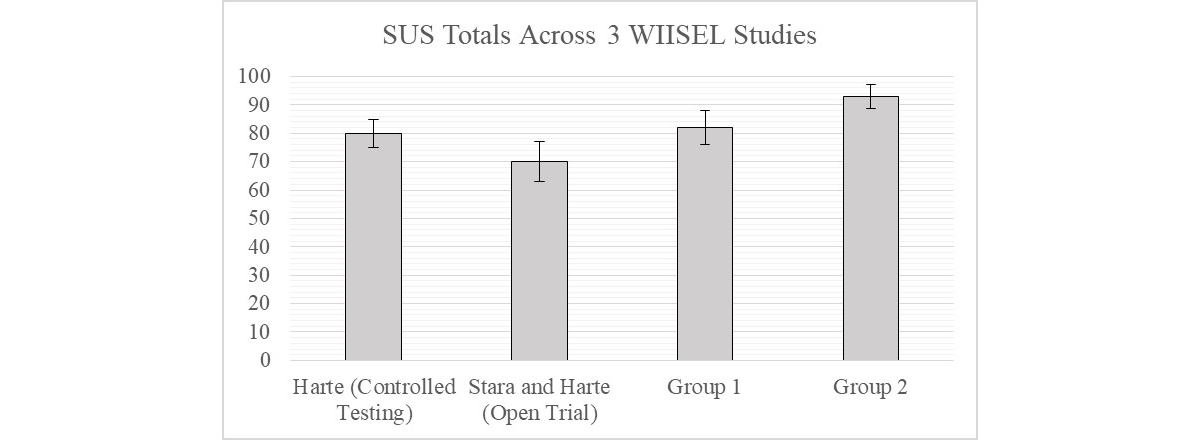
Comparison of System Usability Scale (SUS) scores from various assessments of the user experience of the WIISEL system. WIISEL: Wireless Insole for Independent and Safe Elderly Living.

When comparing the SUS outcomes to previous studies of the WIISEL system, we can see how dramatic the effect of the supplementary smartphone training on the Group 2 participants in this study really was. In Figure 11, we can see that Group 1 exhibited similar SUS results to the participants who took part in a controlled usability test [24] and an open trial [32]. In both of these studies, the participants received no supplementary smartphone training. The Group 2 participants scored significantly higher than the other three groups.

**Table 7:** Regression analysis of satisfaction measure against predictors such as technology experience, perceived usefulness, perceived ease of use, and age.

Group	System Usability Scale (SUS) measures	Technology category (experience)	Perceived usefulness	Perceived ease of use	Age
1 (No extra training)	Overall SUS	0.33	0.47	0.61^a^	0.12
	Usability subscale	0.27	0.19	0.6^b^	0.34
	Learnability subscale	0.33	0.66^b^	0.63^b^	0.51
2 (Extra training)	Overall SUS	0.35	0.57^a^	0.59^a^	0.19
	Usability subscale	0.33	0.69^b^	0.07	0.11
	Learnability subscale	0.18	0.133	0.82^c^	0.43

^a^*P*<.10.

^b^*P*<.05.

^c^*P*<.01.

**Table 8:** Regression analysis of satisfaction measure against performance metrics. SUS: System Usability Scale.

Group	SUS measures	Time taken	Cues required	Errors made	After Scenario Questionnaire
1 (No extra training)	Overall SUS	0.29	0.47	0.7^a^	0.634^b^
	Usability subscale	0.37	0.48	0.8^c^	0.63^b^
	Learnability subscale	0.07	0.78^c^	0.4	0.39
2 (Extra training)	Overall SUS	0.32	0.84^c^	0.44	0.42
	Usability subscale	0.1	0.7^b^	0.55^a^	0.21
	Learnability subscale	0.62^b^	0.54^a^	0.305	0.58^a^

^a^*P*<.10.

^b^*P*<.05.

^c^*P*<.01.

## Discussion

### Review of Findings

This study aimed to understand how supplementary smartphone training could enhance an older adult’s user experience of a smartphone-based connected health system (WIISEL). The results show that overall Group 2 (Extra Training) had a more positive user experience with the WIISEL system according to the statistically significant difference observed in the SUS (and its subscales) scores between the two groups at the end of the 5-day use period. We observed that in the first 3 days of WIISEL use, Group 2 outperformed Group 1 (No extra training) in key usability categories such as errors committed, cues required, and ASQ scores. No significant difference was observed in task times between each group. It was observed that by the fourth day of use, both groups were recording similar performance metrics, implying that there was a ceiling effect, above which no extra smartphone training could have any significant influence on WIISEL use performance. On the basis of the evidence presented in the Results section, we can conclude that providing the users with extra systematic training on the device had a highly positive effect on user experience.

### Observed Problems

We observed a number of recurring problems encountered by the older adult users in each group. These problems occurred with more frequency in Group 1, which resulted in poorer usability metric outcomes. We grouped these problems into 3 categories—touch sensitivity, touch quality or accuracy, and user interface feedback. Within the first category, the users encountered problems when they held buttons for too long which would either deactivate the button press or initiate a secondary undesired function of the button. For example, holding a keypad letter for too long would input a number or symbols instead of letters. A related problem was observed when users were scrolling through a menu, such as in the settings page. Heavy touches while scrolling resulted in the user unintentionally entering an option (Multimedia Appendix 4). The user would then quickly become lost as they would not recognize the screen they were now presented with.

Users unfamiliar with touch screens had a tendency to leave their touch finger hovering near the screen when they were not interacting with the screen, causing unintentional and sometimes unnoticed screen presses. Unintentional touches also occurred if the user gripped the phone incorrectly. Problems within the second category, touch quality or accuracy, included inaccurate button striking and poor quality striking. For example, there was tendency to aim too low when attempting to strike a button, usually owing to the angle at which the user held the screen (Multimedia Appendix 5). This, at times, led to excessive tapping, where the user would rapidly tap the screen in the hope of hitting the button correctly, leading them to unintentionally press a button in close proximity or to inadvertently press a button on the next screen. Finally, inadequate or unrecognized feedback on screen caused problems for some users. For example, many touch screen elements do not look like traditional buttons with clearly marked borders. This caused problems with striking accuracy. Sometimes users did not recognize the subtle changes in color or form that indicated a button had been successfully pressed. Other buttons had strange shapes which the user did not recognize (Multimedia Appendix 5), such as a triangle or an arrow for a send button, which led to hesitation and confusion.

### Comments on Usability Metrics

The regression analysis performed on the usability metrics and how they affected the overall usability outcome from the SUS show that increased errors made and cues required were related to lower overall SUS scores. Increased ASQ scores (indicating greater task satisfaction) were related to higher SUS scores. Task completion times showed a weak to moderate relationship with SUS scores. This could be explained by the fact that many of the required tasks were not time intensive and had a very linear path toward the desired goal. Participants could not really “get lost” within a task or deviate too much from the optimum task path. Most participants who took longer to complete tasks did so because they were simply progressing at that pace. Therefore, task completion time may be a good indicator of overall usability and may depend on the context of the interaction and tasks involved.

With regard to the relationship between technology adoption predictors such as age, technology experience, and PU/PEoU on usability outcomes, we found no strong link for age and technology experience category although there was a moderate link for PU and PEoU.

### Limitations and Recommendations Going Forward

Our methodology meant that the smartphone-specific training was maintained in parallel with the first 3 days of WIISEL training for Group 2 (see Table 3). There is a concern that this concurrent exposure, rather than implementing a cut-off where the smartphone-specific training ceased before Group 2 began using the WIISEL app, affected the outcome of the WIISEL usability data for Group 2 owing to the group having more total smartphone exposure time during the WIISEL app exposure. However, this approach was chosen such that the participant could achieve the benefit of the training they received on days −2 and −1, by recalling what they had learned from day 0 onward and receiving the appropriate guidance to optimize the training [33]. It is in this context that the participant achieves the benefit of the extra training, thereby improving their user experience and increasing their PEoU with the WIISEL system. We understand that for this training approach to be further operationalized, it may need to be streamlined or indeed undergo some structural changes. However, given the context of the study, we felt that our approach worked for the groups in question and allowed us to properly observe and measure their progress with the smartphone and the WIISEL app.

Regarding the specific problems we observed, we can make some instructional recommendations based on our training experience. These are presented in Textbox 1 and are presented within the categories of problems we identified in section Observed Problems.

Further to the specific recommendations for training older adults outlined in Textbox 1, we can make some more general recommendations. First, when approaching the older adult population, it is important to consider any cultural resistance and concerns that could act as hindrance for the uptake of technology-enabled health care. In particular, security, intrusiveness, lack of control, confidentiality, and usability issues can lead to a lack of trust in such technologies. We can assume that some cases of technology rejection are because of a lack of proper, scaffolded (scaffolded learning of technology is the use of progressive steps to allow the learner to gain independence in their use of the technology), and systematic introduction to the new technology [34,33]. This planned exposure, which should come in the form of appropriate training, must achieve the desired positive impact within a short window in order for technology acceptance rather than technology rejection to occur. The training must not only overcome apprehensions about how difficult the technology is to use but must also overcome any misconceptions about how useful the technology is. Older adults, as with many other user groups, overwhelmingly reject technology when there is unclear evidence of personal benefit or improvement in quality of life [35-37]. A key strategy of the training is to build and increase the user’s perception of and trust in the technology. Although instructional activities can take the form of written materials, computer-based programs, or face-to-face communications, our experience in this study shows the benefit of short, yet intensive, periods of task-based learning with direct corrective feedback. This approach may have applications in other domains such as mobile learning in education [38].

**Recommendations for introducing smartphones to older adults.**

Category and Solution or Recommendation

**Touch sensitivity**

Instruct the user to

- Touch and not press the screen. Instruct the user to strike deliberately and with the pad of the fingertip, not the finger nail (Multimedia Appendix 6).
- Carry out slow deliberate scrolls rather than quick stabbing motions.
- Remove fingers completely from the screen area when not interacting and teach them to hold the phone by the rails rather than with the digits wrapped around.
- When moving the textbox cursor, use light, slow, deliberate movement of the index finger.

**Touch quality or accuracy**

Instruct the user to

- Aim for the top portion of the button when striking (Multimedia Appendix 7).
- Hold the phone parallel to their eye line.
- Say the word “smartphone” (or similarly long word) after a button press before attempting to press it again if a button does not respond immediately.
- Avoid touching the tops or bottoms of the screen when swiping.
- Avoid fingers of the holding hand coming in contact with the touch screen surface.

**User interface feedback**

Teach users about

- The different types of touch elements on the screen which may not look like traditional buttons. For example, triangles or arrows for sending messages.
- Teach users to recognize the subtle feedback signals used to indicate button presses, such as slight color changes and button jumps.
- Teach users to recognize “wait” feedback such as tail chasers, loading bars, and hourglasses.

### Conclusions

In this paper, we have discussed significant findings from a usability design study where a training intervention was developed and tested to introduce elderly users with limited or novice technology experience to a smartphone-based connected health care system. Our findings show the importance of properly introducing—in a scaffolded and systematic fashion—older adults to technology to improve their technology acceptance and enhance their user experience. Through our research in this specific context, we feel that it is possible to build or increase trust in connected, mobile, and wearable health technology through the design and deployment of meaningful instructional activities. Although there currently does not exist a structured training methodology for mHealth with older users, the methods and lessons learned in this paper can be used to conceptualize, design, and implement appropriate, bespoke training strategies. We understand that effort and time will not always be available to carry out training on a prolonged basis; however, scaffolded and structured intervention is necessary to ensure successful adoption of useful mHealth technology by elderly users. In addition to informing our future work, we hope the development of the WIISEL system and the guidelines and geragogical (geragogy is a theory which argues that older adults are sufficiently different that they warrant a separate educational theory) activities enumerated here will be widely used for those designing and developing connected health devices and infrastructure for older adults. More importantly, the involvement of end users is a key strategy for recognizing and removing barriers and mitigating design limitations, but our research has shown that this must be carefully planned to influence, drive, and refine systematically the iterative development of connected, mobile, and wearable health care technologies. We think this paper provides a useful enumerative approach to plan and conduct usability evaluations of smartphone apps and to gather user experience validation data, particularly in the domain of education and learning.

## Appendix

Multimedia Appendix 1 Flowchart showing how each participant is classified in terms of mobile technology capability.

Multimedia Appendix 2 WIISEL user manual.

Multimedia Appendix 3 Phone user manual.

Multimedia Appendix 4 (A) The user is scrolling down to access the WIISEL option in the menu; (B) The user is tapping the screen too hard while scrolling and unknowingly presses the Earth app option (the white dot indicates the strike location); (C) The user is now suddenly in an unfamiliar screen and is at risk of pressing further buttons within this option as their hand may still be carrying out a scrolling action.

Multimedia Appendix 5 (A) The user attempts to strike the “Done” button but aims too low (white dot indicates the location of the strike); (B) During smartphone training, there were times when users felt that feedback on the screen was not appropriate; in this example, the user struggles to find the send button to send the text message (the horizontal triangle).

Multimedia Appendix 6 Finger form when touching actuation areas.

Multimedia Appendix 7 Striking accuracy.

## Abbreviations

ASQ: After Scenario Questionnaire
PEoU: perceived ease of use
PU: perceived usefulness
SUS: System Usability Scale
WIISEL: Wireless Insole for Independent and Safe Elderly Living
