# Supplementary figures and images for: Enhancing Home Health Mobile Phone App Usability Through General Smartphone Training: Usability and Learnability Case Study
Source: JMIR Hum Factors. 2018 Apr 26;5(2):e18. doi: 10.2196/humanfactors.7718 (PMC5945986; doi:10.2196/humanfactors.7718)

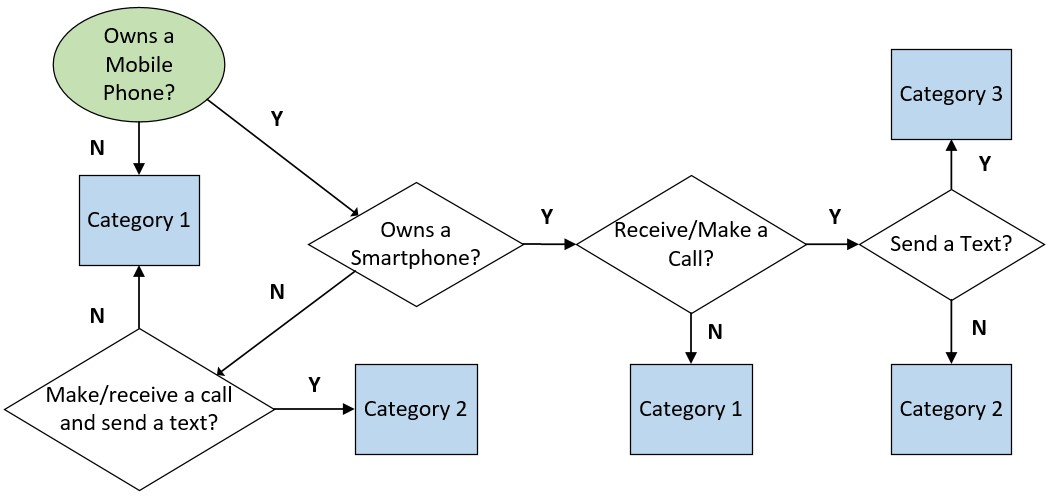

Supplement: Multimedia Appendix 1 [file humanfactors_v5i2e18_app1.png]

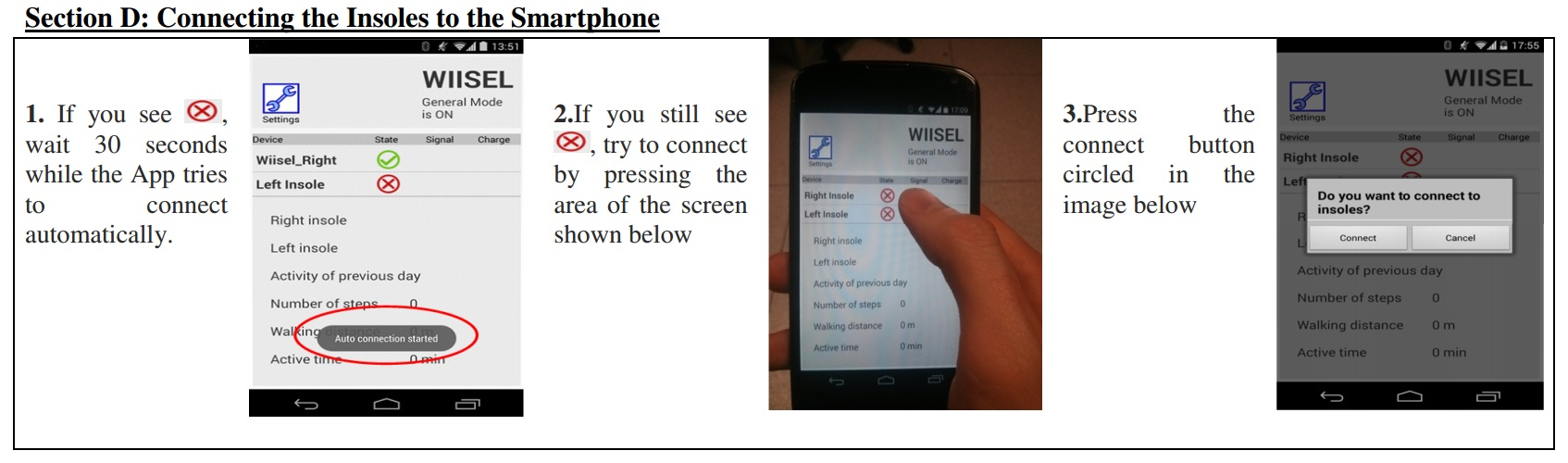

Supplement: Multimedia Appendix 2 [file humanfactors_v5i2e18_app2.png]

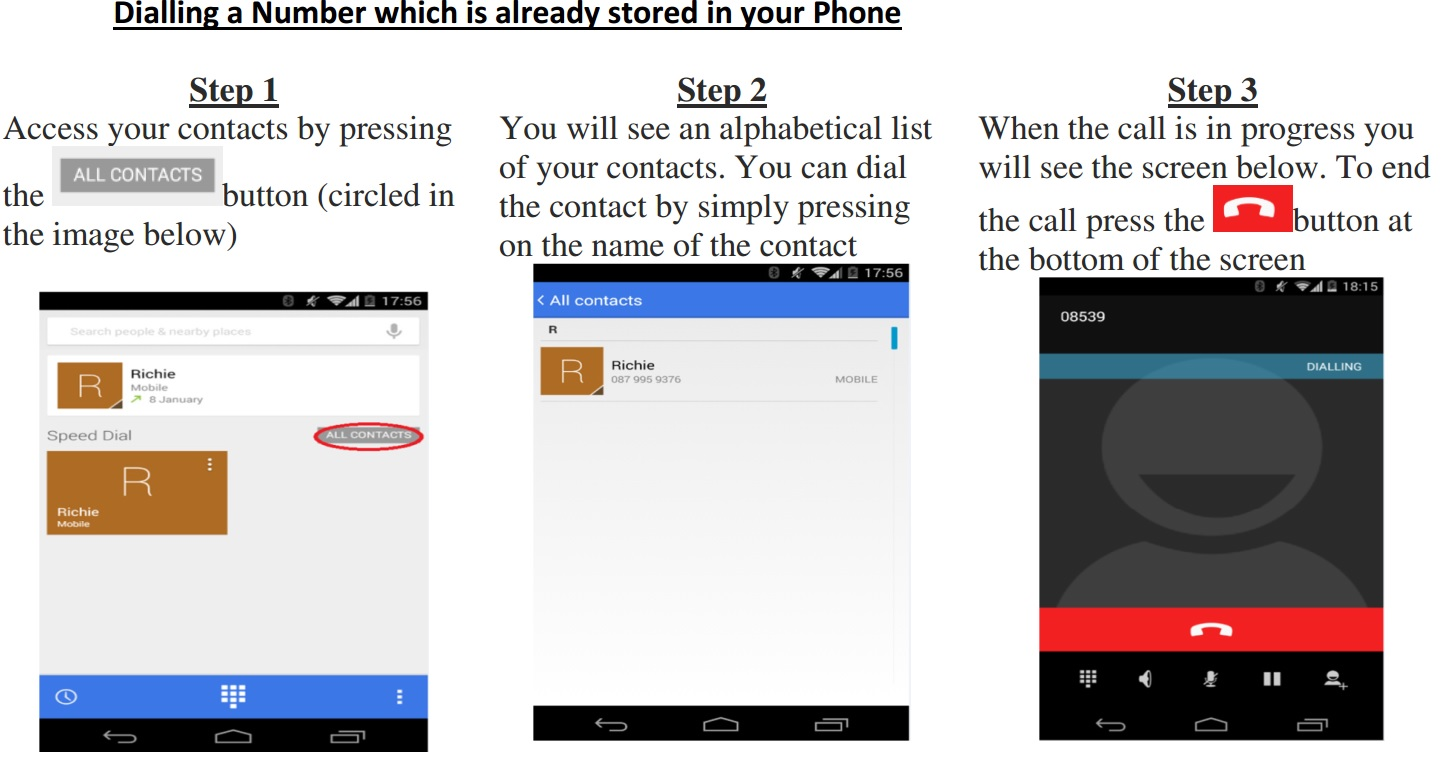

Supplement: Multimedia Appendix 3 [file humanfactors_v5i2e18_app3.png]

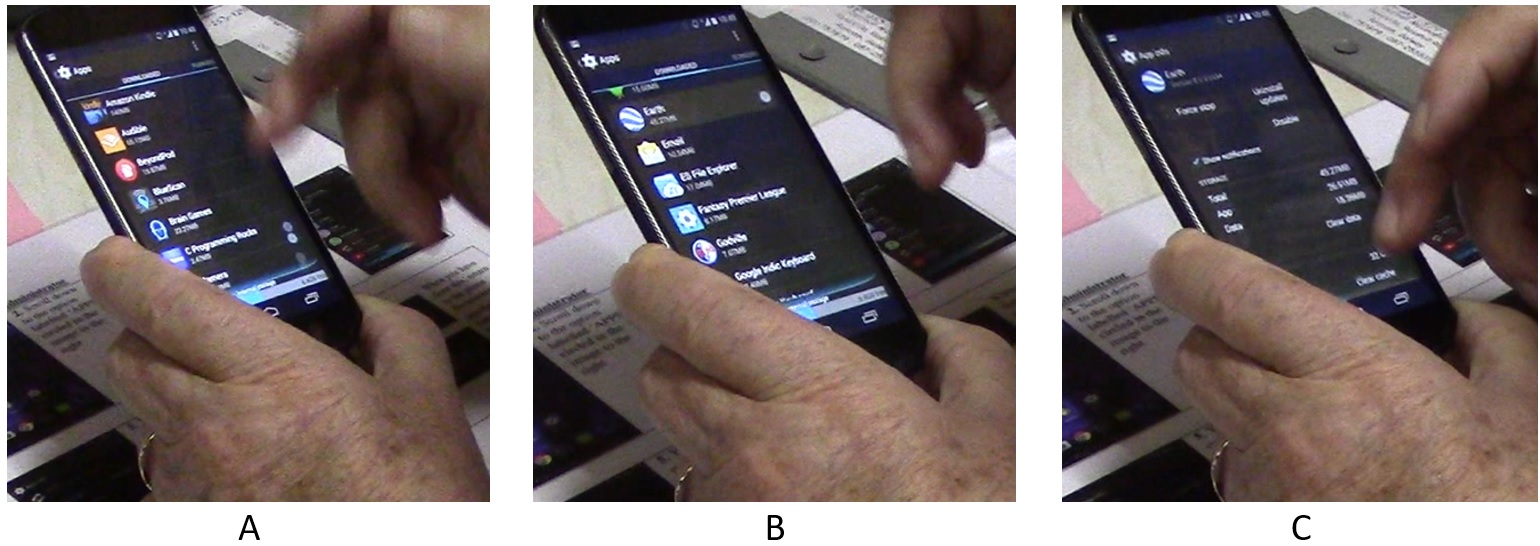

Supplement: Multimedia Appendix 4 [file humanfactors_v5i2e18_app4.png]

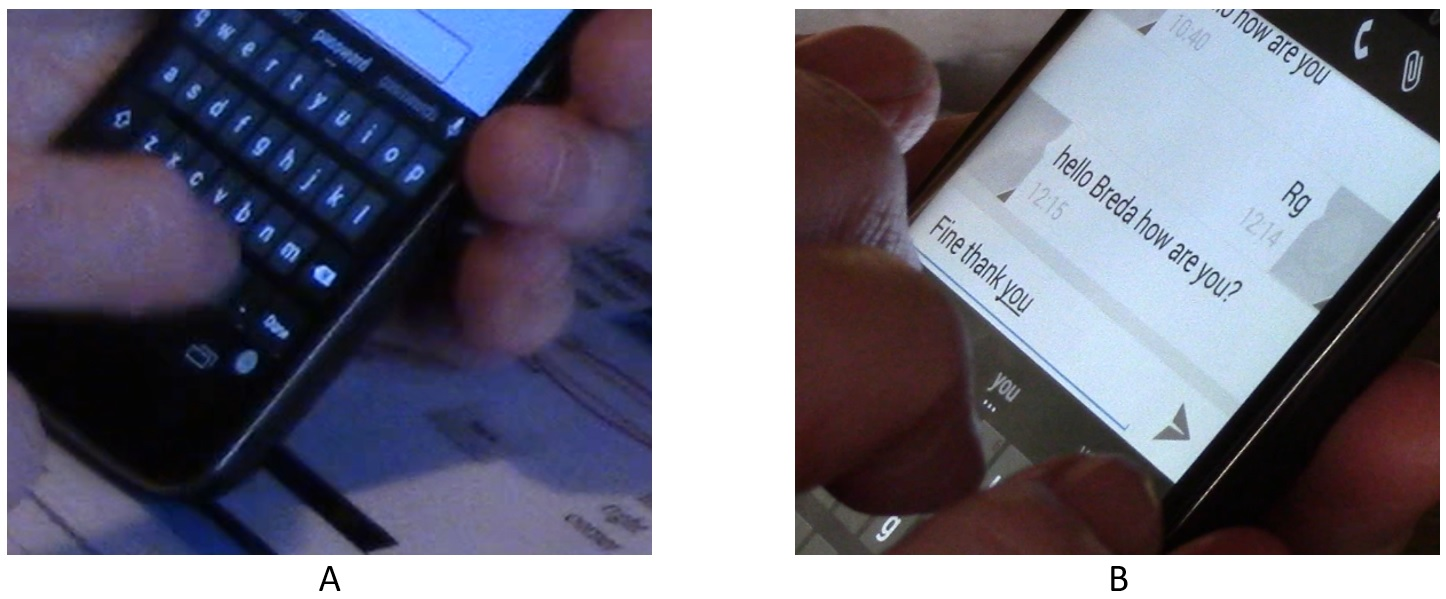

Supplement: Multimedia Appendix 5 [file humanfactors_v5i2e18_app5.png]

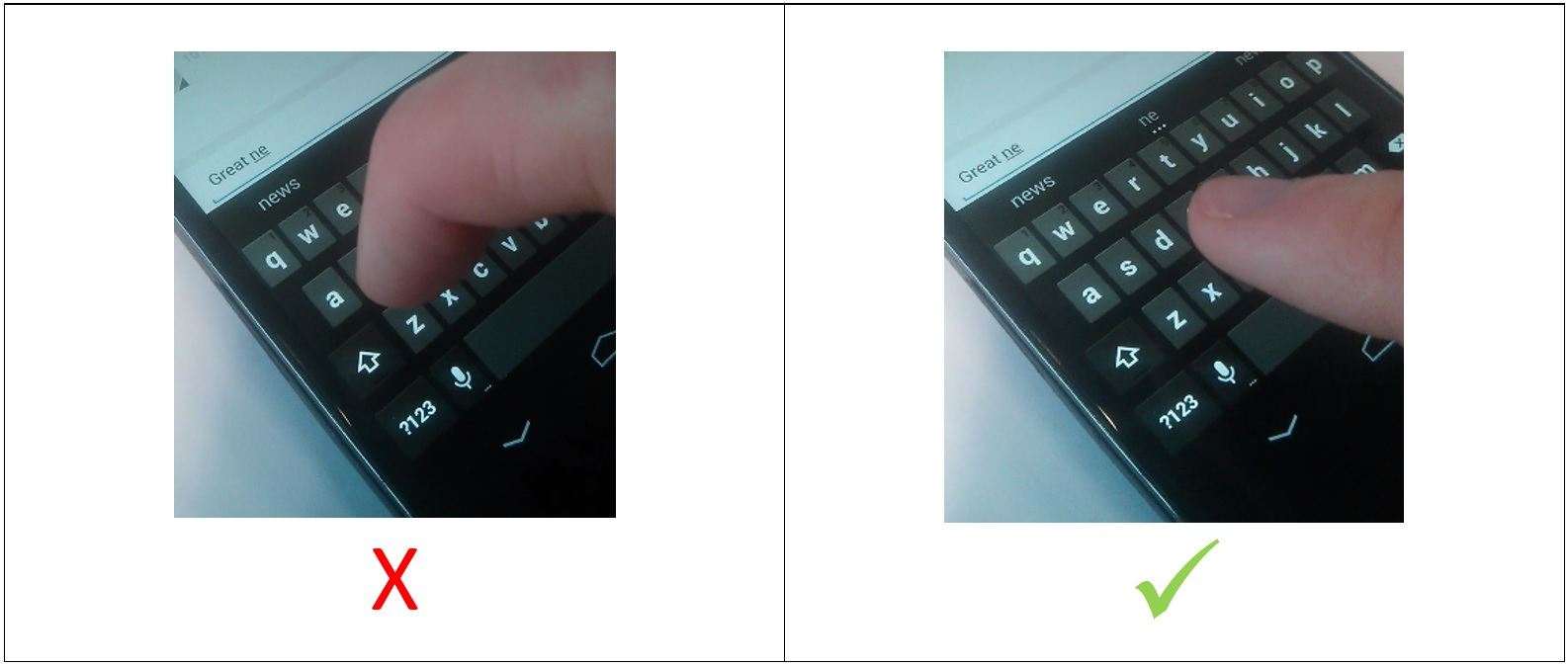

Supplement: Multimedia Appendix 6 [file humanfactors_v5i2e18_app6.png]

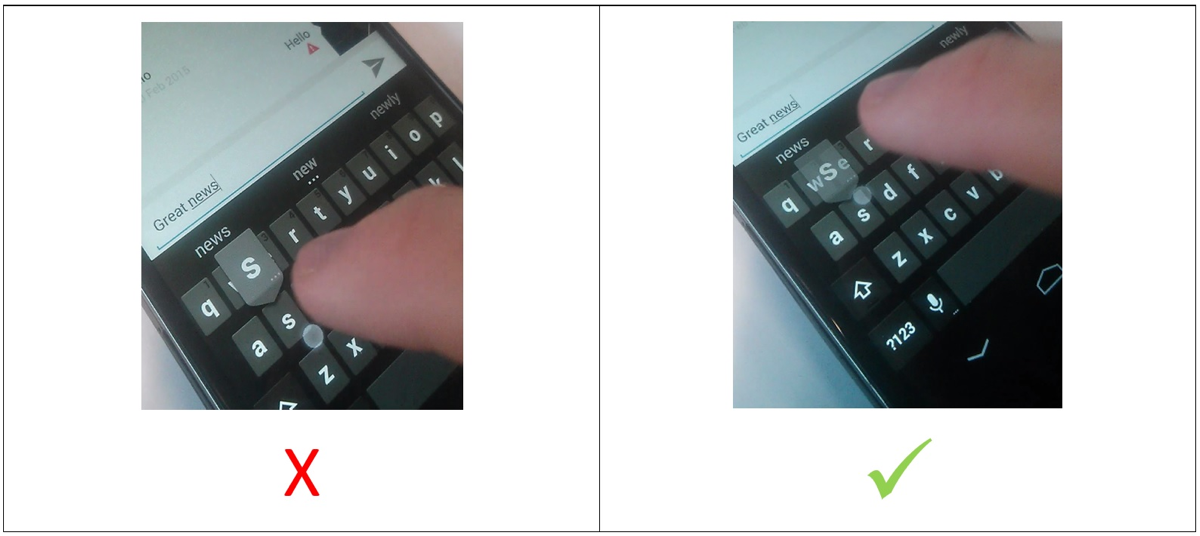

Supplement: Multimedia Appendix 7 [file humanfactors_v5i2e18_app7.png]
